# The Treatment of Tubercular Disease of the Joints

**Published:** 1893-01-07

**Authors:** 


### BRISTOL.

### The Treatment of Tubercular Disease of Joints.

Has the comparatively recently acquired knowledge of the pathology of tubercular disease been any help to us in the treatment of tubercular joint lesions? We know that the exciting agent is a bacillus; can we then in any way deal with the primary cause? The injection of antiseptics into the interior of joints has been often advocated, and several times tried, but has not come into general favour as a method of treatment? Probably the chemical substance used has not been strong enough, or has not come in contact with a sufficient surface of the diseased tissue. Very strong solutions would of course be too corrosive to inject freely. The tuberculin treatment cannot be considered on the same lines; it intensified the destructive action in the tissue, and did not injure the bacillus, and as we all too well know, has been a terrible disappointment. Whether the treatment which Dr. Harrison has lately introduced to the notice of the profession, will prove of any use in joint disease, has yet to be shown. He has had some very good results in cases of lupus. His method consists in the application to the tissues first of a weak acid solution, and then a solution of hyposulphite of ioda, nascent sulphurous acid being formed, which acts injuriously on the bacilli. The solutions might be injected into the pulpy tissue in joints, though of course where the disease began in the bones it could not be brought into contact with the tissues primarily affected.

We have not yet advanced to such scientific therapeutics in the treatment of joint disease, that we can attack the bacillus in its stronghold with known success. What then can we do? In all healthy tissues there is a continual warfare being waged between the tissues and the bacilli which have formed a nidus in them. Put the tissues in the best possible condition to carry on the struggle, and we do a great deal for the patient. Now, locally, rest is one of the essential conditions of successful battle, a principle of treatment recognised long before the bacteriological origin of tubercular joint disease was discovered. The tubercular lesions are attended with chronic inflammatory changes about the joint, and rest subdues these inflammatory conditions, and hence its importance, for there seems reason to think that such chronic inflammatory changes favour the success of the bacilli rather than of the tissues.

Another condition which is very favourable to the growth of the bacillus and the advance of the disease is sepsis. Once let a tubercular joint become septic by the bursting of an abscess or after a partial operative removal of the disease, and the advance of the disease is much accelerated. Hence no threatening joint abscess must on any account be allowed to burst; if it threaten to do so it must be antiseptically opened, but as a rule some more radical measures have to be taken.

In writing of the treatment of the disease by injections of antiseptics into the tissues of the joint, we did not mention the treatment of tubercular joint disease by the injection of iodoform into the joint cavity, because iodoform is known to have very slight power of injuring the bacilli; at any rate, in the presence of oxygen. Yet it has been very largely used in cases where abscess has formed, injected as an emulsion, after the aspiration of the abscess, and after arthrectomies and excision, and an iodoform gauze has been recommended as the best dressing for tubercular joint sinuses, stuffed well into the sinus. Success in these cases seems to suggest that it may act better as a germicide in the absence of oxygen.

Now, the great question we have to consider in each individual case of joint disease is how long we ought to persevere with rest, and when we ought to take operative measures? Of course, if the case is going on well, we shall be content with rest, and any other measures we may take to subdue the chronic inflammatory conditions present, such as counter-irritation. We do not consider any attempt to deal with the disease by arthrectomy, on the ground that there is a risk of general tubercular infection, justifiable, for in the first place we can never be sure of getting rid of every fragment of diseased tissue in the joint, and we may by opening up veins in the diseased part lead to that very infection we are operating to avoid: and in the second place the joint tuberculosis is often not the primary lesion, though we can discover no other, but may be secondary to tubercular bronchial glands. Again, general tubercular infection is not common, considering the frequency of tubercular joint disease, and when it does occur it often follows excision of the hip in the old way, where no attempt was made to clear out, as far as possible, all the tubercular tissue.

There are some conditions of tubercular joints which are, perhaps, better treated by operation than rest at the beginning—when the disease is limited to a part of the soft tissues about the joint, and as yet has not involved the whole synovial membrane, a condition not very rare. Here we act wisely in dealing radically with the localised disease—*i.e.*, by a partial arthrectomy, and we may in this way save the rest of the joint. In cases where the disease begins in one part of the articular end of the bone, we may also sometimes save the joint by its early removal. This has been done even in the case of the hip by removal of sequestra from the neck of the femur.

Does suppuration in a tubercular joint, or, more correctly speaking, breaking down of the tubercular material, necessitate arthrectomy or excision? Mr. Barker regards signs of abscess about the hip as an indication for excision; and cases which suppurate have a much higher mortality than those in which abscess does not form; but Mr. Watson Cheyne and Professor Lister's statistics clearly show that cases in which the abscesses are opened antiseptically and kept aseptic do very well. Hence we cannot take suppuration in connection with tubercular joint disease, as *per se*, an indication always to excise. But with more thorough methods of arthrectomy, such as Mr. Watson Cheyne describes in his recent lectures on the treatment of tubercular joint disease at the College of Surgeons, the tendency of modern surgery seems to be to perform this operation when abscess forms, rather than simply to open the abscess. We say to perform this operation, but in adults, and in the case of the hip even in children, excision with thorough removal of the diseased soft tissues may be preferable. Excision is not an operation for children. Even if we do not cut away the epiphyseal junction, we so damage it by the changes in the bone subsequent to the operation, that we interfere greatly with its growth, and may lead to serious shortening of the limb. It is better to freely scrape the tubercular foci out of the ends of the bones in arthrectomy. In the hip, however, we must remove the head of the femur in order to get access to the joint at all. In adults, where the growth of the bone is complete, it may be better to get rid of the articular ends by excision where there is extensive bone disease, and osseous ankylosis is desirable as in the knee. But in excision we must cut away as much of the tubercular pulpy tissue as in simple arthrectomy, and not merely remove the bone, as was generally done before the advantage of more thorough removal of the diseased tissue was recognised.

